# Assessment of Donor Derived Cell Free DNA (dd-cfDNA) at Surveillance and at Clinical Suspicion of Acute Rejection in Renal Transplantation

**DOI:** 10.3389/ti.2023.11507

**Published:** 2023-10-13

**Authors:** Evangelos Mantios, Vassilis Filiopoulos, Pantelis Constantoulakis, George Liapis, Angeliki Vittoraki, Silvia Casas, Smaragdi Marinaki, John N Boletis

**Affiliations:** ^1^ Department of Nephrology and Kidney Transplantation, School of Medicine, General Hospital of Athens Laiko, National and Kapodistrian University of Athens, Athens, Greece; ^2^ “Genotypos” Science Laboratory of Molecular Genetic and Cytogenetics, Athens, Greece; ^3^ Pathology Department, School of Medicine, General Hospital of Athens Laiko, National and Kapodistrian University of Athens, Athens, Greece; ^4^ Immunology Department, National Tissue Typing Center, General Hospital of Athens “G. Gennimatas”, Athens, Greece; ^5^ CareDx Inc., Brisbane, CA, United States

**Keywords:** dd-cfDNA, kidney allograft, transplantation, rejection, biomarker

## Abstract

In our prospective, unicenter cohort study, we collected blood samples from 30 newly kidney transplanted patients, at month 1, 2, 3, and 5 for dd-cfDNA analysis, along with creatinine/eGFR and DSA monitoring, and from 32 patients who underwent an indication biopsy and whose dd-cfDNA levels were measured at the time of biopsy and 1 month afterwards. Fourteen of 32 (43.8%) patients in the biopsy group were diagnosed with TCMR and 5 of 32 (15.6%) with ABMR. Dd-cfDNA proved to be better than creatinine in diagnosing rejection from non-rejection in patients who were biopsied. When a dd-cfDNA threshold of 0.5% was chosen, sensitivity was 73.7% and specificity was 92.3% (AUC: 0.804, 0.646–0.961). In rejection patients, levels of dd-cfDNA prior to biopsy (0.94%, 0.3–2.0) decreased substantially after initiation of treatment with median returning to baseline already at 1 month (0.33%, 0.21–0.51, *p* = 0.0036). In the surveillance group, high levels of dd-cfDNA (>0.5%) from second month post-transplantation were correlated with non-increasing eGFR 1 year post-transplantation. The study used AlloSeq kit for kidney transplant surveillance for first time and confirmed dd-cfDNA’s ability to detect rejection and monitor treatment, as well as to predict worse long-term outcomes regarding eGFR.

## Graphical Abstract

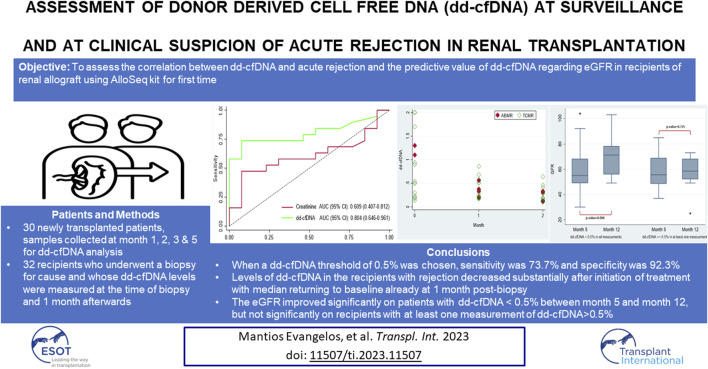

## Introduction

Rejection, antibody-mediated, and T-cell mediated, remains the first cause of death-censored allograft loss in kidney recipients [1, 2]. Despite the standardization of needle biopsy for rejection diagnosis, it is rarely used for surveillance due to its cost, logistics, potential complications, and patient discomfort. Only 17% of US centers conduct surveillance biopsies, and another 21% do so on a selective basis [3]. Donor-derived cell-free DNA (dd-cfDNA) has been proposed as a non-invasive marker for transplant rejection, not only in kidney [4–9], but also in lung [10, 11] and heart transplants [12, 13], since it may itself trigger inflammation and thus add insult to injury [14, 15]. In renal transplant recipients who developed *de novo* donor specific antibodies (dnDSAs), a rise in dd-cfDNA > 0.5% occurred a median of 91 days preceding detection of dnDSAs [16]. The first large multicenter trials aiming to compare dd-cfDNA measurements with the molecular phenotype of kidney transplant biopsies [17], as well as short patient series trying to enhance the use of dd-cfDNA information to guide clinical practice and immunomodulation decisions [18] have recently been published, while additional interventional studies are in progress [19].

We launched a prospective study for the assessment of dd-cfDNA in renal transplantation, which is an observational longitudinal cohort with 62 patients and used Alloseq kit, that was implemented locally for dd-cfDNA testing in order to provide information about the clinical performance of the biomarker in surveillance and rejection detection for first time. By using AlloSeq cfDNA assay, study aims to evaluate the correlation between dd-cfDNA values in plasma and DSA formation, as well as between the dd-cfDNA measurements and histopathology reporting, based on “for cause” renal biopsy. Additionally, we aimed to examine the long-term relationship between elevation in dd-cfDNA and estimated glomerular filtration rate (eGFR).

## Patients and Methods

### Study Population

A total of 30 adult kidney transplant recipients in one transplant center were monitored with dd-cfDNA testing at month 1, 2, 3, and 5 post-transplant (surveillance group). The initial surveillance group included 39 patients, 9 of whom underwent an indication biopsy during the surveillance period and therefore were “transferred” to the biopsy group. The biopsy group was consisted of 32 renal recipients who were biopsied for cause and were monitored with dd-cfDNA prior to biopsy and 1 month afterwards. Data was collected between 1 November 2020, and 20 January 2022. The study performed in accordance with international standards, and it did not form part of a broader study. The patients were managed prospectively as standard of care without dd-cfDNA in the context of post-transplant care, with dd-cfDNA data captured being retrospectively examined. Using the center’s medical records, we determined clinical events (e.g., rejection, infection) and routine laboratory tests (creatinine, DSAs). Participants had to meet the inclusion criteria of the study; male or female, aged 12 years or above, recently transplanted and willing and able to give informed consent for participation in the trial and to comply with all trial requirements. Pregnant women, recipients of multiple organs, patients with significant hepatic impairment or short life expectancy, monozygotic twins and patients who had previously received bone marrow transplants were not allowed to participate in the study. None of the recipients were excluded from participation. Polyomavirus infection did not constitute an exclusion criterion from the study.

### dd-cfDNA Testing

Venous blood was collected in Cell-Free DNA BCT tubes (Streck, La Vista, NE) and plasma isolated according to manufacturer’s instructions (Streck) used for analysis. An analysis sample of 240 dd-cfDNA measurements was collected from 62 patients for this study. The cell-free DNA was extracted from the isolated plasma by using QIAamp Circulating Nucleic Acid Kit (Qiagen, Hilden, Germany) and then 10 ng inputed for library preparation with AlloSeq cfDNA kit following assay manual documentation IFU084 version 6.0, September 2021 provided by the manufacturer (CareDx Pty, Fremantle, WA, Australia). The resulting amplified products were sequenced on the MiSeq sequencing system (Illumina, San Diego, CA), and sequencing data was analyzed with AlloSeq cfDNA software version 1.0 (CareDx Pty). The AlloSeq cfDNA is a commercially available next-generation sequencing (NGS)-based assay that identifies the fraction of donor-specific cfDNA by analyzing 202 targeted single-nucleotide polymorphisms (SNPs), chosen to have genome-wide coverage (equally distributed), multiethnicity coverage and high uniformity. Genetic relationship between donor and recipient was entered into AlloSeq cfDNA Software, and the algorithm adjusts % the dd-cfDNA calculation accordingly. Assuming a reporting range of <50% for kidney post-transplant, no recipient or donor samples were provided, and AlloSeq cfDNA software algorithm assumed the minor represented cfDNA fraction as the donor fraction to calculate the % dd-cfDNA. In addition to % dd-cfDNA, AlloSeq cfDNA QC metrics for all loci, mean coverage, uniformity, and locus count were monitored.

### Diagnosis of Graft Dysfunction and Biopsy-Defined Rejection

Results of for-cause kidney transplant biopsies were recorded. Among the indications for for-cause biopsy were changes in creatinine, worsening proteinuria, the development of dnDSA, or a combination of these factors (Table 1). A single pathologist blinded to dd-cfDNA results assessed biopsy reports for study analysis. Interpretations of biopsy results were made in accordance with Banff 2019 classification scheme [20]. Antibody-mediated rejection (ABMR) group included also mixed rejection cases. Borderline cases were captured and categorized in the T cell–mediated rejection (TCMR) group.

**TABLE 1:** Indications for biopsy in the biopsy group.

Indications for biopsy	*N* = 32
sCr increase	15
Non satisfactory sCr decrease (early post-Tx period)	6
Extended DGF (>20 days)	1
BK viremia + sCr increase	5
Deterioration of proteinuria	1
*De novo* DSAs	4

Other concomitant pathologic diagnoses, such as calcineurin inhibitor toxicity, glomerulopathy, or acute tubular injury or acute tubular necrosis (or both) were classified as no rejection. Rejection treatment decisions were made following the center’s clinical protocol. As part of the surveillance group of 30 newly transplanted patients and of the group of those who had a biopsy, all dd-cfDNA levels were collected, along with eGFR changes and dnDSAs.

### Statistical Analyses

Distributions of categorical variables were summarized through absolute and relative (%) frequencies. For continuous variables, mean and standard deviation (SD) were used for the normally distributed variables, while median and interquartile range (IQR) for the non-normally distributed ones. Statistical analysis was performed by either Wilcoxon rank-sum (Mann-Whitney U), Wilcoxon Signed Rank or Kruskal-Wallis H nonparametric statistical tests (non-normally distributed continuous variables). In addition, ROC analysis and a two-way repeated-measures analysis of variance (ANOVA) were performed. All statistical analyses were performed using Stata version 16.0 program. The level of statistical significance was set at 0.05.

Univariable and multivariable exact logistic regression models were used to identify factors associated with rejection for patients with biopsy. Rejection was determined as the binary dependent variable [outcomes: rejection/non-rejection; T cell-mediated rejection (TCMR)/non-rejection] and dd-cfDNA in month 0, age, gender, ABO incompatibility, DSAS preformed, DSAS *de novo*, days after transplantation and Crossmatch B flow as possible explanatory (independent) variables. The significance level was set equal to 0.10 for the univariable analyses and equal to 0.05 for the multivariable analyses. Odds ratios (ORs) and 95% confidence intervals (95% CI) are reported.

#### dd-cfDNA and eGFR Analysis

Kidney function was determined by eGFR calculated using the Chronic Kidney Disease-Epidemiology Collaboration (CKD-EPI) equation. Dd-cfDNA and eGFR for each month was assessed. There were two categories of patients: those with a high dd-cfDNA (any measurement above 0.5%) and those with a low dd-cfDNA (all measurements below 0.5%). A two-way repeated-measures analysis of variance (ANOVA) was performed for the analysis.

## Results

The demographics of the 62 patients enrolled in our study depict a population of high immunological risk (Table 2). An ABO incompatible transplant was performed on one patient out of five in both the surveillance and biopsy groups. It was noted that 23.3% of patients who were newly transplanted had preformed DSAs, while 43.8% of patients who were biopsied had preformed DSAs. Plasmapheresis and intravenous immunoglobulin were administered prior to surgery to one of every three recipients either due to DSAs or because of ABO incompatibility. Among the biopsy group, the rejection diagnosis was identified in 19 out of 32 patients (59.4%), with 14 of the 19 being classified as TCMR. In three patients, ABMR was diagnosed, while in two recipients, mixed rejection was detected, which was also classified as ABMR.

**TABLE 2:** Descriptive statistics of (i) newly transplanted patients (*n* = 30) and (ii) patients with biopsy (*n* = 32).

Variable	Newly transplanted patients (*n* = 30)	Patients with biopsy (*n* = 32)
Mean age [years, (SD)]	46.5 (10.8)	41.5 (14.3)
Primary disease [*n*, (%)]		
DN	1 (3.3)	1 (3.1)
Glomerulonephritis	12 (40.0)	13 (40.6)
Nephronophthisis	0 (0.0)	2 (6.3)
Obstructive uropathy	2 (6.7)	5 (15.6)
Other	0 (0.0)	1 (3.1)
PKD	5 (16.7)	4 (12.5)
Unknown	10 (33.3)	6 (18.8)
Median years of haemodialysis (IQR^a^)	1.5 (0.0, 8.0)	1.5 (0.5, 7.5)
Transplantation [*n*, (%)]		
Deceased donor	8 (26.7)	11 (34.4)
Living donor	22 (73.3)	21 (65.6)
Donor (relation) [*n*, (%)]^b^ ^,^ ^c^		
Husband	6 (27.3)	2 (9.5)
Wife	3 (13.6)	3 (14.2)
Father	2 (9.1)	1 (4.8)
Mother	9 (40.9)	13 (61.9)
Brother	0 (0.0)	1 (4.8)
Sister	0 (0.0)	1 (4.8)
Aunt	2 (9.1)	0 (0.0)
Mean age of donor [years, (SD)]	55.4 (15.0)	55.1 (15.5)
Donor history [factors; *n*, (%)]		
0	13 (43.3)	12 (37.5)
1	12 (40.0)	14 (43.8)
2 or 3	5 (16.7)	6 (18.8)
ABO incompatibility [*n*, (%)]		
No	24 (80.0)	25 (78.1)
Yes	6 (20.0)	7 (21.9)
DSAS preformed [*n*, (%)]		
No	23 (76.7)	18 (56.2)
Yes	7 (23.3)	14 (43.8)
DSAS de novo [*n*, (%)]		
No	30 (100.0)	28 (87.5)
Yes	0 (0.0)	4 (12.5)
Crossmatch B flow [*n*, (%)]		
No	24 (80.0)	26 (81.3)
Yes	6 (20.0)	6 (18.7)
Crossmatch T flow [*n*, (%)]		
No	29 (96.7)	30 (93.8)
Yes	1 (3.3)	2 (6.2)
RTX [*n*, (%)]		
No	19 (63.3)	22 (68.8)
Yes	11 (36.7)	10 (31.2)
PLEX + IVIG [*n*, (%)]		
No	20 (66.7)	20 (62.5)
Yes	10 (33.3)	12 (37.5)
ATG [*n*, (%)]		
No	27 (90.0)	25 (78.1)
Yes	3 (10.0)	7 (21.9)
Median days after transplantation (IQR)	—	106.5 (19.0, 185.0)
Rejection [*n*, (%)]		
No	—	13 (40.6)
ABMR^d^		5 (15.6)
TCMR^e^		14 (43.8)
Prednisone pulses [*n*, (%)]		
No	—	15 (46.9)
Yes		17 (53.1)
PLEX [*n*, (%)]		
No	—	28 (87.5)
Yes		4 (12.5)
ATG [*n*, (%)]		
No	—	30 (93.8)
Yes		2 (6.2)
Leflunomide [*n*, (%)]	—	
No		29 (90.6)
Yes		3 (9.4)
Eculizumab [*n*, (%)]	—	
No		31 (96.9)
Yes		1 (3.1)

^a^ IQR, interquartile range.

^b^ Newly transplanted patients: *n* = 22.

^c^ Patients with biopsy: *n* = 21.

^d^ ABMR, antibody-mediated rejection.

^e^ TCMR, T cell-mediated rejection.

### Association of dd-cfDNA Levels and Acute Rejection Events

**FIGURE 1:**
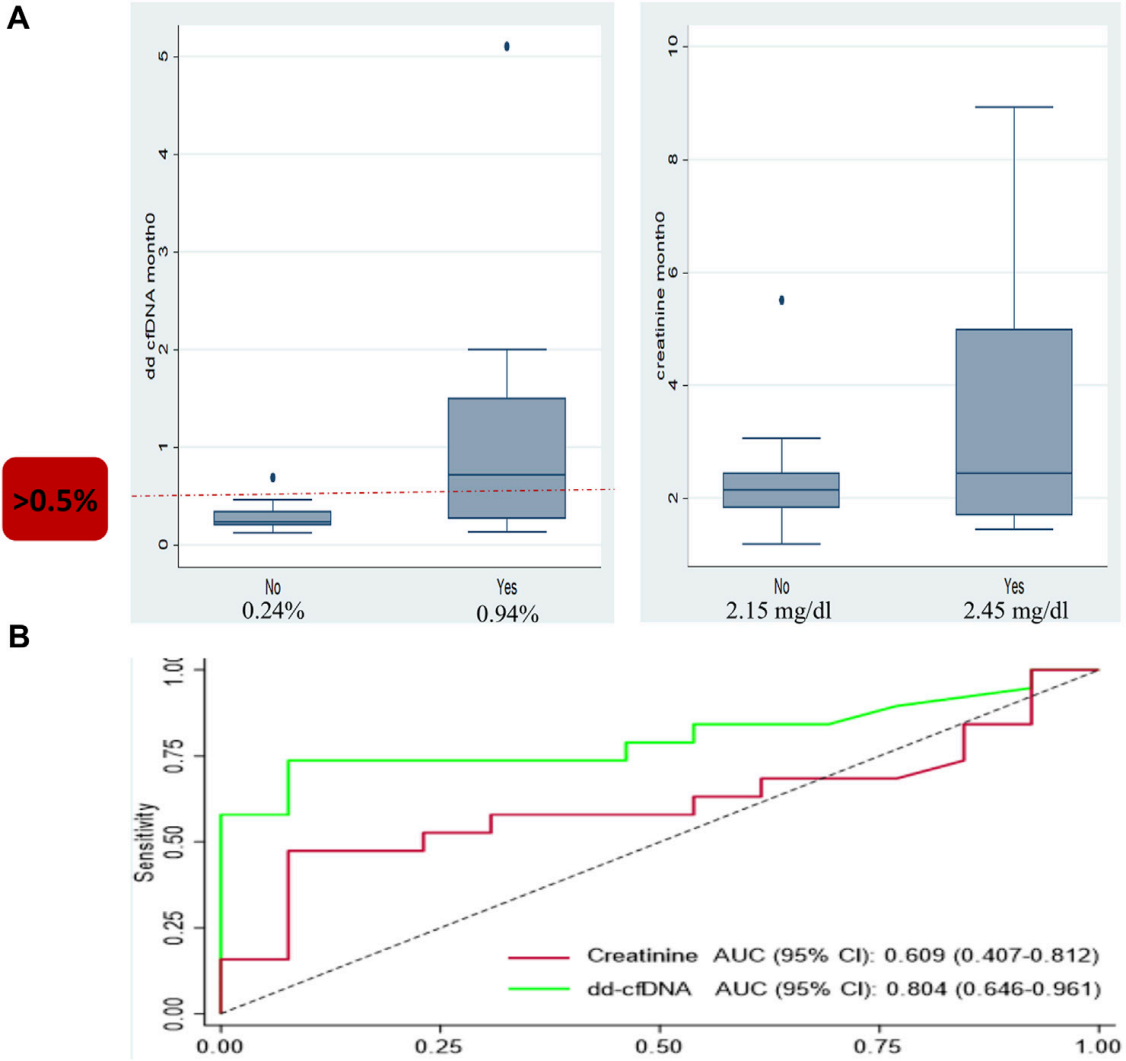
Box and whisker plot and ROC analysis showing the median donor-derived cell-free DNA (dd-cfDNA) and creatinine levels observed in patients with and without allograft rejection. **(A)** Box and whisker plot for dd-cfDNA (left) showing a median of 0.24% seen in patients with no rejection and 0.94% in patients with allograft rejection; *p* = 0.004. Box and whisker plot for creatinine (right) with a median creatinine of 2.15 mg/dL in patients with no rejection versus 2.45 mg/dL in patients with allograft rejection; *p* = 0.3. **(B)** The ROC analysis for dd-cfDNA: area under the receiver-operating characteristic curve (AUROC) 0.804. The ROC analysis for creatinine: AUROC 0.609.

Using 32 for cause biopsies from 32 patients with biopsy-paired dd-cfDNA results, the association between dd-cfDNA levels and any allograft rejection status was evaluated. Even though changes in serum creatinine make up the largest proportion of reasons for a biopsy in our study, there was no statistically significant difference in the median creatinine in patients with a no rejection biopsy (2.15 mg/dL; interquartile range [IQR]: 1.82–2.44 mg/dL) and patients with Banff-defined rejection (2.45 mg/dL; IQR: 1.70–4.98 mg/dL); *p* = 0.3 (Figure 1). The AUROC for creatinine was 0.609 (95% CI: 0.407–0.812). In comparison, the median dd-cfDNA level among patients with a no rejection biopsy was 0.24% (IQR: 0.20%–0.34%), which was significantly lower than the median dd-cfDNA in patients with biopsies demonstrating defined cellular or antibody-mediated rejection (0.94%; IQR: 0.30%–2.0%); *p* = 0.004. The AUROC for all rejection dd-cfDNA was 0.804 (95% CI: 0.646–0.961). The Youden’s index for dd-cfDNA was 0.58%. When a dd-cfDNA threshold of 0.5% was chosen, sensitivity was 73.7% and specificity was 92.3%.

ABMR was diagnosed in 5 biopsies. Among these patients, compared to non-rejection patients, the median dd-cfDNA was 13%; *p* < 0.001. TCMR was diagnosed in 14 biopsies. Patients with TCMR, compared to nonrejection patients, had a median dd-cfDNA value of 0.52%; *p* = 0.038.

In terms of discrimination, dd-cfDNA was effective for distinguishing among biopsies that show no rejection or any rejection. However, when it exceeded a specific threshold, it could rise the possibility for any type of rejection. Patients with dd-cfDNA higher than 0.5% had more than 25 times higher odds of rejection compared to those with dd-cfDNA lower than 0.5% (*p* < 0.001) and more than 12 times higher odds of TCMR compared to those with dd-cfDNA lower than 0.5% (*p* = 0.031) (Tables 3, 4).

**TABLE 3:** Multivariable exact logistic regression estimates using rejection as the binary outcome variable (outcomes: rejection/non-rejection).

Explanatory variable	Adjusted odds ratio	95% Conf. Interval	*p*-value
dd-cfDNA (in month 0)			
*<0.5%	—	—	—
≥0.5%	25.57	(3.44, +Inf)	**<0.001**

*Reference category.

Patients with dd-cfDNA higher than 0.5% had a more than 25 times higher odds of rejection compared to those with dd-cfDNA lower than 0.5% (*p* <0.001).

**TABLE 4:** Multivariable exact logistic regression estimates using TCMR as the binary outcome variable [outcomes: T cell-mediated rejection (TCMR)/non-rejection].

Explanatory variable	Adjusted odds ratio	95% Conf. Interval	*p*-value
dd-cfDNA (in month 0)			
*<0.5%	—	—	—
≥0.5%	12.35	(1.18, 746.10)	**0.031**

Univariable and multivariable exact logistic regression models were used to identify factors associated with rejection for patients with biopsy. Rejection was determined as the binary dependent variable [outcomes: rejection/non-rejection; T cell-mediated rejection (TCMR)/non-rejection] and dd-cfDNA in month 0, age, gender, ABO incompatibility, transplantation, DSAS, DSAS *de novo*, days after transplantation and Crossmatch B flow as possible explanatory (independent) variables.

*Reference category.

Patients with dd-cfDNA higher than 0.5% had a more than 12 times higher odds of TCMR compared to those with dd-cfDNA lower than 0.5% (*p* = 0.031).

### Monitoring Anti-Rejection Treatment

**FIGURE 2:**
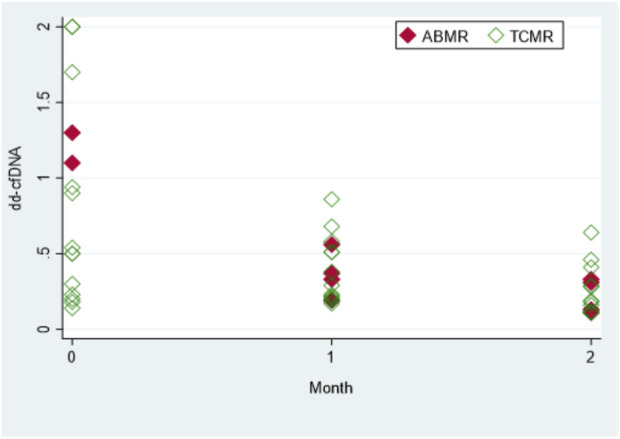
The dd-cfDNA kinetics with anti-rejection treatment. Total of 15 patients with biopsy and rejection [antibody-mediated rejection (ABMR) or T-cell mediated rejection (TCMR)]. Values shown are at month 0 (time of biopsy and diagnosis), and at 1 and 2 months (after rejection treatment was initiated). For the sake of clarity, four patients with high levels of dd-cfDNA (>2.0%) were excluded from the graph presented. Each diamond represents a biopsy specimen.

Dd-cfDNA kinetics were evaluated in 19 recipients diagnosed with rejection (Figure 2). In order to achieve a longer monitoring period, dd-cfDNA levels were also measured 2 months after biopsy in 15 out of 19 rejection recipients. Levels of dd-cfDNA before biopsy (0.94%; IQR: 0.3–2.0) decreased substantially after initiation of treatment already at first month (0.33%; IQR: 0.21–0.51); *p* = 0.0036. The difference was even more significant when comparing median dd-cfDNA levels at month 2 (0.19%; IQR: 0.12–0.33) to median levels at month 0 (*p* = 0.0007).

According to our study, the median value of dd-cfDNA for 30 surveillance patients from the first 5 months post-transplantation was 0.23% (IQR: 0.18%–0.36%). Moreover, nine transplant recipients who were initially enrolled in the surveillance group had median dd-cfDNA of 0.33% (IQR: 0.24%–0.37%) before being referred for a graft biopsy and being ‘transferred’ to the biopsy group. These findings suggest that median dd-cfDNA levels returned to baseline levels already at the first month after anti-rejection treatment, while dd-cfDNA levels at month 2 were similar to the median dd-cfDNA levels of the surveillance group.

### Association of dd-cfDNA Elevation and eGFR Progression

**FIGURE 3:**
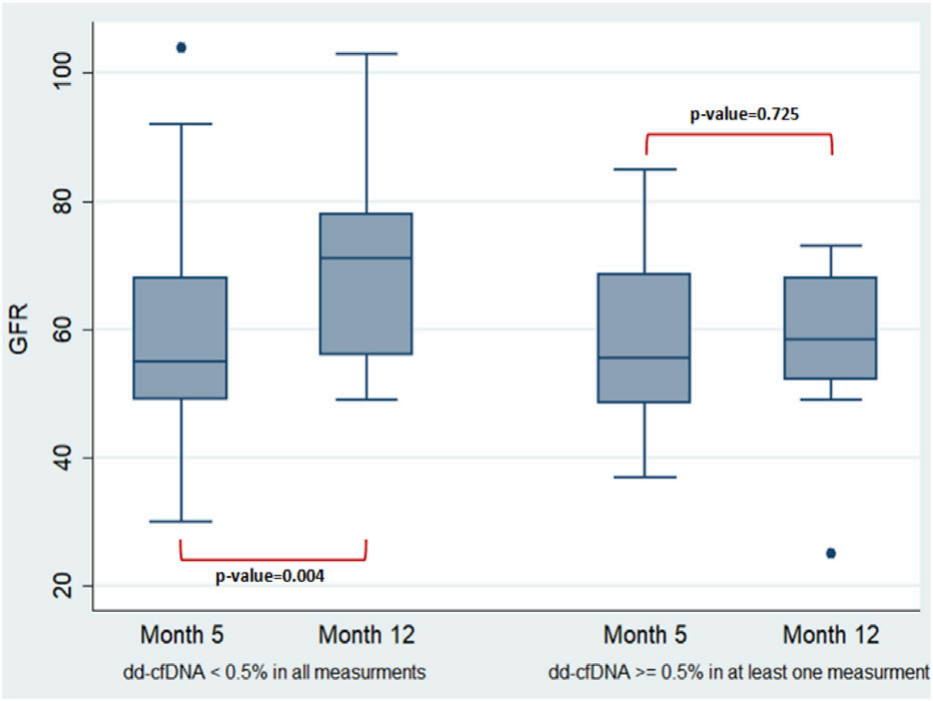
Two-way repeated-measures ANOVA performed to examine the effect of group (newly transplanted patients were grouped according to the percentage of dd-cfDNA and the number of measurements) and time on the eGFR revealed non-significant main effect of group (*p* = 0.2235), non-significant main effect of time (*p* = 0.2008) and non-significant interaction between factors (the effects of group and time on eGFR) (*p* = 0.0652). In more detail, the analysis determined that the mean value of eGFR has not been significantly different between the groups and the timepoints. A difference of the mean value of eGFR between the timepoints was observed only for those with dd-cfDNA < 0.5% (*p* = 0.004). The two-way repeated-measures ANOVA analysis with the Greenhouse-Geisser correction (performed to check if the data do not meet the compound symmetry assumption) confirmed the previous estimates.

In the surveillance group of the 30 newly transplanted recipients, an effort was made to assess how the elevation of dd-cfDNA affects changes in eGFR 1 year post-transplantation. The two-way repeated-measures ANOVA was run on the eGFR at month 5 and 12 in two groups of 22 (dd-cfDNA < 0.5% in all measurements—month 1 excluded) and 8 (dd-cfDNA ≥ 0.5% at least in one measurement—month 1 excluded) newly transplanted patients. Groups were defined according to the percentage of dd-cfDNA (cut-off point = 0.5%) and the number of measurements (Figure 3). A difference of the mean value of eGFR between month 5 and month 12 was observed for patients with dd-cfDNA < 0.5% (*p* = 0.004) compared to recipients with at least one high measurement of dd-cfDNA (≥0.5%) (*p* = 0.725), whose eGFR did not seem to rise that efficiently 1 year post-transplantation. However, the mean value of eGFR has not been significantly different between the two groups in month 12.

### Correlation of Alterations in dd-cfDNA Over Time With Indication Biopsies

As mentioned above, nine of the 32 renal recipients who underwent a biopsy had been enrolled in the surveillance group at the beginning of the study but were shifted to the biopsy group after an indication for a for cause biopsy was received. All these recipients were biopsied after the second month post-transplantation. As a result, nine patients had at the end of the study at least two monthly dd-cfDNA measurements prior to the biopsy event. We decided to compare the first two measurements of these recipients to the first two dd-cfDNA measurements of the 30 surveillance patients who managed to complete 5 months post-transplantation without the need of a for cause biopsy.

**FIGURE 4:**
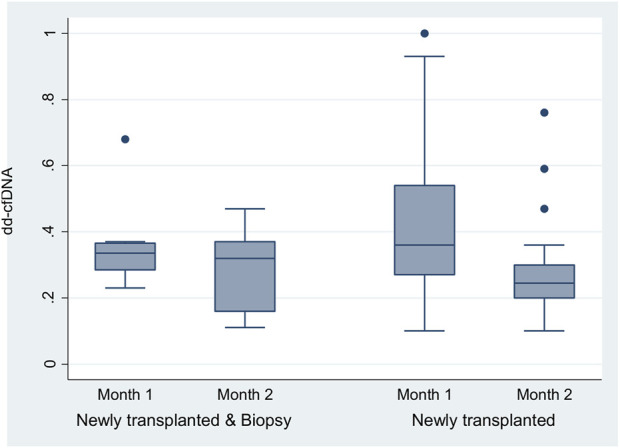
The two-way repeated-measures ANOVA that was run to examine the effect of group (newly transplanted patients and newly transplanted patients who had experienced biopsy) and time on the dd-cfDNA revealed non-significant main effect of group (*p* = 0.5480), a significant main effect of time [F (1, 36) = 5.72, *p* = 0.0221] and non-significant interaction between factors (the effects of group and time on dd-cfDNA) (*p* = 0.3083). In more detail, the analysis determined that the mean value of dd-cfDNA has not been significantly different between the groups, but has been significantly different between the timepoints (month 1 and 2). The difference of the dd-cfDNA between the timepoints was observed mainly for the newly transplanted patients (*p* = 0.001). The two-way repeated-measures ANOVA analysis with the Greenhouse-Geisser correction confirmed the previous estimates.

The two-way repeated-measures ANOVA performed on the dd-cfDNA at month 1 and 2 in two groups of 9 newly transplanted with biopsy and 30 newly transplanted patients showed a greater reduction of dd-cfDNA in patients who did not need a biopsy (*p* = 0.001) compared to those who needed one the first months post-transplantation (Figure 4).

### Relationship Between dd-cfDNA Level and Identification of dnDSAs

None of the 30 surveillance recipients developed dnDSAs the first year post-transplantation and only 4 of the 32 patients who performed a biopsy did so. Due to these circumstances, an analysis was not possible between dd-cfDNA and DSA formation.

## Discussion

Our study is the first conducted in Europe and also the first one of the Greek cohort of kidney transplant patients to investigate the clinical performance of dd-cfDNA in both surveillance and for-cause biopsies, by using AlloSeq kit, a laboratory product that can be implemented and operated, without the need to send samples to a centralized service. There have been larger studies that have derived similar conclusions, but these used centralized service tests for dd-cfDNA and primarily included US cohorts [6, 16]. This commercially available *in vitro* diagnostics kit was implemented locally for dd-cfDNA testing and investigated Greek cohort for the first time. Several studies have also examined dd-cfDNA’s diagnostic potential in different areas of kidney transplantation using the Alloseq cfDNA kit. Mayer et al. assessed the diagnostic value of dd-cfDNA in the diagnosis of ABMR based on Alloseq, as an adjunct to the detection of DSA [21], as well as its ability to differentiate rejection from BK nephropathy [22]. Moreover, the researchers used AlloSeq to investigate whether dd-cfDNA levels are affected by clazakizumab, a promising anti-rejection treatment [23]. Other authors assessed AlloSeq’s value as a surveillance tool after reduction of immunosuppression in order to accomplish seroresponse in transplant recipients who had not responded in previous COVID-19 vaccinations [24, 25]. AlloSeq cfDNA assay was also used in other studies to examine different analytical techniques for the quantification of donor-derived cell-free DNA in plasma and urine [26, 27].

Using the AlloSeq assay, we measured dd-cfDNA in renal transplantation as a percentage rather than an absolute measurement. It is hotly debated whether absolute quantification is superior to fractional measurement in discrimination of rejection. A cross-sectional study in Australia compared diagnostic performance of dd-cfDNA (cp/mL) and dd-cfDNA (%), and similar results were obtained for composite diagnosis of ABMR [30]. In a German prospective cohort, the comparison of % versus cp/ml dd-cfDNA results were not significantly different regarding NPV and PPV, even though the AUC for cp/ml was significantly higher [28]. A single-Center Cohort in California proposed combining cp/ml and fractional results, but the suggested superior diagnostic performance was based only on the results of a cohort of 9 rejection cases [9]. Furthermore, the multi-centric Trifecta study did not find a significant difference in dd-cfDNA performance between reporting with cp/ml and reporting with fractions. AUC increased only slightly when cp/ml and fraction were combined [29]. On the contrary, R. Gohn et al. found that absolute quantification of dd-cfDNA did not provide any additional discriminating power over dd-cfDNA fraction for detection of allograft rejection [17]. Moreover, while % cut-offs have been observed to be consistent across cohorts and sites, it is important to note that cp/mL are difficult to standardize across sites: 21 and 12 cp/mL (used by Whitlam JB et al. [30]) vs. 52 cp/mL (proposed by Oellerich M et al. [28]) and vs. 78 cp/m (used at Trifecta [29]).

It has been reported in the recent ADMIRAL study that patients with clinical ABMR had significantly higher levels of dd-cfDNA (2.2% versus 0.34%) [16], whereas in our cohort the ABMR median was even higher (13.0% versus 0.24%). Statistically significant increases in dd-cfDNA were also observed in patients with clinically evident TCMR (0.52% vs. 0.24%) compared to patients without clinical evidence of rejection. The results of a recent meta-analysis, which included six studies that used a 1.0% threshold for dd-cfDNA to diagnose rejection, indicated a diagnostic odds ratio of 8.18 for the biomarker [31]. The high median value of ABMR, as well as the high odds ratio for rejection in general when dd-cfDNA exceeded 0.5% in our cohort [25], was attributed to the small sample size of the study and some really high dd-cfDNA measurements in 3 out of 5 ABMR patients, 2 of whom had stopped their immunosuppression and ended up in allograft loss as a consequence of these devastating rejection episodes. Some high measurements in the TCMR group were also the reason for the high odds ratio for TCMR [12] despite the anticipated, compared to the literature, TCMR median value (0.52%). Despite the general perception, based on several studies [32–34], that dd-cfDNA is less effective in diagnosing TCMR than ABMR, the high odds ratio for TCMR in our study comply with the latest work of Aubert et al. [35], who included 1,210 biopsies in 992 patients and concluded that higher levels of dd-cfDNA were observed for ABMR and TCMR or both compared to other diagnoses. Sigdel et al. also reported higher dd-cfDNA fractions in TCMR patients [36].

Given the small number of participants, we decided to include the 2 patients diagnosed with borderline rejection in the TCMR group (14 patients overall), keeping in mind although the heterogeneous injury within this diagnosis, as Stites et al. [37] proved by risk-stratifying recipients with TCMR1A and borderline rejection depending on their dd-cfDNA level prior to biopsy. As a result of the small sample size, no TCMR analysis was conducted according to TCMR grade, which remains a current knowledge gap in literature: nine studies included in the meta-analysis of Wijtvliet V. et al [34], who did not find higher dd-cfDNA levels in TCMR patients compared to recipients without rejection at indication biopsy, reported no dd-cfDNA fractions for different grades of TCMR, making a distinction between dd-cfDNA fractions in low versus high grades of TCMR impossible. Due to the high heterogeneity of TCMR and the lack of differentiation between low grade and high grade TCMR in the published dd-cfDNA studies, further research is required on dd-cfDNA values for different grades of TCMR.

It was calculated that our optimal threshold for dd-cfDNA using AlloSeq cfDNA assay in order to discriminate rejection from non-rejection was 0.58%, which complies with the ADMIRAL study, whose Youden Index for dd-cfDNA was 0.69%, while the AUROCs in the two studies when using the same threshold were similar [16].Former studies have considered 1.0% as the appropriate threshold [5, 15, 36]. In order to increase its sensitivity, more studies which will combine dd-cfDNA with other biomarkers such as urinary chemokines, may be of great interest in the future.

The delta between serial dd-cfDNA was also associated with increased possibility for an indication biopsy, suggesting that dd-cfDNA alterations can be an alarming sign for the allograft quiescence. Using the dd-cfDNA as an indicator, Anand et al. [38]showed that a 141% increase in dd-cfDNA is associated with abnormal pathology. Our AlloSeq study showed that not only the increase, but also the non-satisfactory decrease in dd-cfDNA in the early post-transplant period can indicate that a patient may require closer monitoring or even invasive procedures in the future.

Wolf-Doty et al. [39] have monitored dd-cfDNA in 35 patients from the DART study who received anti-rejection treatment and concluded that 1 month post-rejection dd-cfDNA levels returned from 0.62% to 0.35%, which was almost the baseline for the non-rejection recipients of the DART study (0.30%). Our AlloSeq study confirms these findings, with median rejection value before biopsy (0.94%; IQR: 0.3–2.0) returning to baseline already at first post-rejection month (0.33%; IQR: 0.21–0.51); *p* = 0.0036.

It was concluded that recipients with low dd-cfDNA levels showed a clear increase in eGFR after month 12 (*p* = 0.004), while those with at least one high dd-cfDNA value excluding first month did not show the same increase. It should be noted, however, that due to the short follow-up period, the mean value of eGFR has not been significantly different between the two groups after month 12. Bu et al. [16] demonstrated a correlation between higher levels of dd-cfDNA and a subsequent decline in eGFR, while Huang et al. [40] stated that as compared to assessing graft survival using only biopsy characteristics alone, the addition of dd-cfDNA to Banff biopsy scores provides a superior prognostic assessment.

It has been demonstrated by Aubert et al. [41] that dnDSAs have a detrimental effect on the graft survival in comparison with preexisting DSAs, while a study by Lionaki et al. [42] reported a link between dnDSAS and reduced allograft survival, even in the absence of clinically evident ABMR. 87 patients from the DART study were identified by Jordan et al. [43]as evidence that the PPV of dd-cfDNA increases when used in combination with dnDSAs. After a 1 year follow-up period, none of our surveillance recipients with serial dd-cfDNA monitoring developed dnDSAs, and only four of the patients who were biopsied had developed dnDSAS. It should be noted that three of the four patients who had dnDSA had ABMR and had levels of dd-cfDNA >1%, while the fourth patient had recurrence of primary FSGS without any rejection and had a low level of dd-cfDNA at diagnosis (0.31%). A small sample size made it impossible to perform any analysis.

Previously mentioned, the major limitation of our study was the small sample size, which prevented the correlation of dd-cfDNA with DSA formation, and in combination with some very high values in ABMR patients, led to large confidence intervals and high diagnostic odds ratios regarding rejection. Nevertheless, these limitations did not affect the AUROC performance or usefulness of the biomarker as a monitoring tool. A longer follow up period could also strengthen the correlation between high dd-cfDNA and worse outcome regarding eGFR over time. Moreover, in view of the evident multifactoral value of considering dd-cfDNA as part of the clinical assessment of the patient, further research is required to determine the optimal monitoring interval. The novelty of this study is that Alloseq dd-cfDNA kit was used locally for dd-cfDNA testing and useful clinical data was provided about how this kit performed in the real-world.

In summary, this report using AlloSeq cfDNA kit for local testing confirms large multicenter service-based trials regarding dd-cfDNA’s validity as a tool to surveil the allograft quiescence, to detect rejection and monitor treatment, as well as to predict outcomes regarding graft survival. However, since dd-cfDNA is positioned to be added within the existing panel of current routine testing rather than be used as single information for taking clinical decision, it is undisputed that further research combining dd-cfDNA with other biomarkers is required to improve our diagnostic tools in relation to allograft rejection.

## Data Availability

The original contributions presented in the study are included in the article/supplementary material, further inquiries can be directed to the corresponding author.
